# Morphological and Pathological Evolution of the Brain Microcirculation in Aging and Alzheimer’s Disease

**DOI:** 10.1371/journal.pone.0036893

**Published:** 2012-05-16

**Authors:** Jesse M. Hunter, Jason Kwan, Michael Malek-Ahmadi, Chera L. Maarouf, Tyler A. Kokjohn, Christine Belden, Marwan N. Sabbagh, Thomas G. Beach, Alex E. Roher

**Affiliations:** 1 The Longtine Center for Neurodegenerative Biochemistry, Banner Sun Health Research Institute, Sun City, Arizona, United States of America; 2 Cleo Roberts Center for Clinical Research, Banner Sun Health Research Institute, Sun City, Arizona, United States of America; 3 Department of Microbiology, Midwestern University, Glendale Arizona, United States of America; 4 Civin Laboratory for Neuropathology, Banner Sun Health Research Institute, Sun City, Arizona, United States of America; Nathan Kline Institute and New York University School of Medicine, United States of America

## Abstract

Key pathological hallmarks of Alzheimer’s disease (AD), including amyloid plaques, cerebral amyloid angiopathy (CAA) and neurofibrillary tangles do not completely account for cognitive impairment, therefore other factors such as cardiovascular and cerebrovascular pathologies, may contribute to AD. In order to elucidate the microvascular changes that contribute to aging and disease, direct neuropathological staining and immunohistochemistry, were used to quantify the structural integrity of the microvasculature and its innervation in three oldest-old cohorts: 1) nonagenarians with AD and a high amyloid plaque load; 2) nonagenarians with no dementia and a high amyloid plaque load; 3) nonagenarians without dementia or amyloid plaques. In addition, a non-demented (ND) group (average age 71 years) with no amyloid plaques was included for comparison. While gray matter thickness and overall brain mass were reduced in AD compared to ND control groups, overall capillary density was not different. However, degenerated string capillaries were elevated in AD, potentially suggesting greater microvascular “dysfunction” compared to ND groups. Intriguingly, apolipoprotein ε4 carriers had significantly higher string vessel counts relative to non-ε4 carriers. Taken together, these data suggest a concomitant loss of functional capillaries and brain volume in AD subjects. We also demonstrated a trend of decreasing vesicular acetylcholine transporter staining, a marker of cortical cholinergic afferents that contribute to arteriolar vasoregulation, in AD compared to ND control groups, suggesting impaired control of vasodilation in AD subjects. In addition, tyrosine hydroxylase, a marker of noradrenergic vascular innervation, was reduced which may also contribute to a loss of control of vasoconstriction. The data highlight the importance of the brain microcirculation in the pathogenesis and evolution of AD.

## Introduction

The number of elderly individuals with neurodegenerative disorders such as Alzheimer’s disease (AD) has expanded dramatically [1]. Alzheimer’s disease is an age-associated multisystemic syndrome. Cardiovascular disease (CVD), the most prevalent cause of morbidity and mortality [2], has long been associated with vascular cognitive impairment (VCI) and represents a potential contributing factor to AD development [3]–[6]. Studies suggest that common cardiovascular-related diseases such as hypertension [7], [8], hypotension [9], atherosclerosis [10], arteriosclerosis [11], stroke [12], coronary artery disease [13] and diabetes [14] are risk factors for VCI and AD. In addition, the major genetic risk factor for AD, possession of the apolipoprotein E (ApoE) ε4 allele [15], [16], is also a recognized risk factor for CVD [10], [17]. Ultrasonography, imaging and biochemical studies have demonstrated that individuals with AD have diastolic dysfunction [18], decreased carotid diastolic velocities [19], decreased mean flow velocities and increased pulsatility indices [19], decreased total and regional cerebral blood flow [20], reduced brain perfusion [21], disturbances of the blood-brain barrier and neurovascular unit alterations [4], [22]. Furthermore, a diminished cardiac output and cardiac index have been associated with aging [23], [24]. Cerebral amyloid angiopathy (CAA) also plays a major role in brain hypoperfusion and dysfunction. Brain perfusion is further damaged by CAA due to a compromised interstitial fluid drainage resulting from the destruction of the perivascular spaces by the accumulation of amyloid [25]. Moreover, hypoperfusion promotes loss of blood flow shear stress, resulting in endothelial cell death and collapse of capillaries contributing to the formation of string vessels [26], [27]. In addition to all these pathological changes, cerebral hypoperfusion also promotes vascular inflammation [4] and the expression of hypoxia-inducible and pro-angiogenic factors [28]. The emergence of anti-angiogenic molecules, including Aβ peptides [29], may counterbalance activation of vascular remodeling and repair functions [4], [30].

Along with the cardiovascular and cerebrovascular diseases that interfere with cerebral perfusion, in AD there is impairment of regional cerebral blood flow which is regulated by the neurovascular units that respond to local biochemical demands [3], [6], [31]–[33]. These regulatory mechanisms of cerebral blood flow are deeply disturbed in AD, resulting in hypoperfusion as elegantly demonstrated by arterial spin labeling [34], [35]. In addition to this regional autoregulation, the brain vasoactive centers are severely injured in AD. Cholinergic projections from the nucleus basalis of Meynert (NBM) extend into the cortex and are a major mediator for vasodilation of cortical arterioles and capillaries. Stimulation of the NBM activates muscarinic and nicotinic acetylcholine receptors in perivascular neurons and astrocytes to release the vasodilator nitric oxide [36]–[40]. Vasoconstriction is mediated in part by catecholaminergic and serotonergic innervation emanating from the locus ceruleus and dorsal raphe nucleus, respectively [41]–[43].

Considering the cumulative consequences of CAA and combined cardiovascular and cerebrovascular disease in AD, it would be expected that non-demented (ND) control individuals have a better conservation of brain microvasculature than those with AD. The presence and abundance of amyloid plaques and CAA increases with age, but not all individuals with high amyloid plaque burdens and CAA develop cognitive impairment. An explanation for this apparent contradiction may be that cognitively normal individuals with high amyloid loads have better cerebrovascular preservation. In order to address these questions, some structural aspects of the microvasculature and its innervation as well as their relationships to brain atrophy were investigated in oldest-old subjects with and without amyloid plaques and with and without dementia. In addition, a ND cohort of younger individuals without amyloid plaques was included for comparison. For the final assessment of the morphological and pathological conditions of the microvasculature among these groups, we combined the available clinical and neuropathological data to generate an overall brain fitness index (BFI) for each individual.

## Materials and Methods

### Human Subjects

Brain specimens were obtained from the Banner Sun Health Research Institute Brain and Body Donation Program [44]. The operations of the Brain and Body Donation Program, including those related to this study, have been approved by the Banner Health Institutional Review Board. All subjects enrolled in the Brain and Body Donation Program sign an informed consent approved by the Banner Health Institutional Review Board. All AD subjects met NINCDS-ADRDA criteria for a clinical diagnosis of probable and possible AD as previously published [45]. Cases examined in this study were selected on the bases of age and the guidelines established by the National Institute on Aging (NIA)-Reagan Institute, to warrant classification as AD, and were not complicated by other neuropathological diagnoses, including Lewy body lesions. The study compared four neuropathologically assessed cohorts **(** **Table 1** **)**: 1) six nonagenarian individuals diagnosed clinically as having AD (cases 10–15) harboring sufficient AD amyloid plaque density to meet NIA-Reagan criteria of “intermediate” or “high” probability that dementia was due to AD; 2) eight nonagenarian individuals (cases 1–8) clinically assessed as ND with sufficient amyloid plaque density to meet NIA-Reagan criteria of “intermediate” probability that dementia, were it present, would be due to AD. These individuals were classified as non-demented high pathology controls (ND-HPC); 3) six nonagenarian individuals (cases 50–55) clinically assessed as ND exhibiting total plaque scores of zero, classified as oldest-old no plaque controls (OO-NPC) and 4) five septuagenarian individuals (cases 60–64) clinically assessed as ND and exhibiting total plaque scores of zero, classified as young-old no plaque controls (YO-NPC). Other AD-related pathologies such as neurofibrillary tangle (NFT) score, Braak stage, CAA, white matter rarefaction (WMR) and ApoE genotype were not considered in the selection of these cases. The complete neuropathological and epidemiological data of the selected cases are included in **Table 1** and the **Table S1**.

**Table 1:** Tabulation of the demographic, clinical and neuropathological parameters.

ID	Expiredage (y)	Gender	PMI (h)	Brainweight (g)	Last MMSE score	ApoEgenotype	Total plaque score	NP density	Total NFTscore	Braak stage	Total WMR score	Total CAA score
10	95	F	3.2	1040	16	3/4	12.2	Freq.	10.0	VI (6)	0	8
11	90	M	14.0	1300	19	3/3	11.0	Mod.	6.3	IV (4)	7	2
12	96	F	3.0	1000	18	3/3	13.8	Mod.	8.0	IV (4)	1	6
13	96	F	3.0	900	5	2/3	10.0	Freq.	15.0	VI (6)	12	12
14	96	F	3.3	960	13	3/4	11.5	Freq.	15.0	VI (6)	10	9
15	92	F	2.8	900	13	4/4	14.5	Freq.	15.0	VI (6)	3	2
Mean	94.2		4.9	1017	14.0		12.2		11.5	5.33	5.5	6.5
ND-HPC												
1	91	M	3.0	1050	-	3/4	10.8	Mod.	5.0	III (3)	0	0
2	100	M	2.5	1160	29	3/3	14.0	Mod.	8.0	IV (4)	1	8
3	90	F	4.3	975	28	3/3	10.5	Freq.	5.0	III (3)	1	0
4	94	M	3.5	1100	27	3/3	15.0	Freq.	10.5	IV (4)	10	9
5	90	F	2.5	966	25	3/3	13.5	Freq.	8.0	IV (4)	2	1
6	92	M	3.2	1300	27	2/4	14.0	Freq.	12.0	V (5)	1	8
7	91	M	4.3	1150	29	2/3	14.5	Mod.	8.5	IV (4)	1	1
8	94	M	2.5	1050	29	3/3	15.0	Freq.	12.0	IV (4)	2	1
Mean	92.8		3.2	1094	27.7		13.4		8.6	3.88	2.3	3.5
OO-NPC												
50	91	F	5.0	1112	-	2/3	0.0	Zero	5.0	III (3)	2	0
51	91	M	2.0	1330	30	3/4	0.0	Zero	7.5	IV (4)	1	0
52	91	F	2.5	1100	30	2/3	0.0	Zero	6.5	IV (4)	1	0
53	91	F	7.3	1100	30	3/3	0.0	Zero	4.0	IV (4)	4	0
54	99	F	3.5	975	29	3/3	0.0	Zero	3.5	III (3)	5	0
55	92	M	2.7	1225	29	3/3	0.0	Zero	8.5	IV (4)	1	0
Mean	92.5		3.8	1140	29.6		0.0		5.8	3.67	2.3	0.0
YO-NPC												
60	75	F	2.8	1110	29	3/4	0.0	Zero	5.0	III (3)	0	0
61	71	M	3.0	1300	26	3/3	0.0	Zero	0.0	I (1)	0	0
62	68	F	2.6	1140	29	3/3	0.0	Zero	3.5	III (3)	4	0
63	65	M	3.5	1400	-	3/3	0.0	Zero	1.0	I (1)	2	0
64	75	M	3.3	1262	-	3/3	0.0	Zero	0.5	I (1)	3	0
Mean	70.8		3.0	1242	28.0		0.0		2.0	1.80	1.8	0.0

**Table 1** **abbreviations and explanations.** AD = Alzheimer’s disease; ND-HPC = non-demented high pathology control; OO-NPC = oldest-old no plaque control; YO = young-old no plaque control; ID = Case identification number, y = years, PMI = post mortem interval, h = hours, Brain weight indicates the brain mass at autopsy, g = grams, MMSE = mini mental state exam, ApoE = apolipoprotein E, NP = neuritic plaque, Freq. = frequent, Mod. = moderate, NFT = neurofibrillary tangle, WMR = white matter rarefaction, CAA = cerebral amyloid angiopathy. String vessels indicates the average number of string vessels from numerous images, TH = tyrosine hydroxylase and TH density indicates the average % area of numerous images of sections stained with an anti-TH antibody. VAChT = vesicular acetylcholine transporter and VAChT vesicles indicates the average number of vesicles per image from numerous images of VAChT stained sections. Gray matter thickness is the distance in pixels from the tissue edge to the nearest area of white matter. Capillary number represents the average number of capillary objects from numerous images of collagen IV stained sections while capillary density represents the average % area covered by capillaries from the same images.

### Neuropathological Evaluation

The neuropathological examination procedures used in this study were previously described in Maarouf et al. [46]. Amyloid deposits, NFT and WMR were visualized by staining 40 µm sections with Campbell-Switzer, Thioflavine-S, Gallyas and hematoxylin and eosin (H&E). The clinicopathological diagnosis of AD was established if cases had an NIA-Reagan rating of “intermediate” or higher, neuritic plaque density moderate or frequent and Braak NFT stage III-VI [47] and were clinically demented. Plaque densities (all plaque types, including diffuse, neuritic and cored, were considered together) were reported numerically as 0, 1, 2 and 3, for none, sparse, moderate and frequent, respectively, using the CERAD templates [48], [49]. Five separate regions were appraised: frontal, temporal, parietal, hippocampal and entorhinal, to render a maximum score of 15. The total NFT score was ranked in the same fashion as described for plaques, again using the published CERAD templates. The Braak stage (I-VI) was estimated in thick sections according the original method described by Braak and Braak [47]. White matter rarefaction was evaluated on one-quarter of hemisphere sections stained by H&E in the frontal, temporal, parietal and occipital lobes. The evaluations were none, mild (less than 25% affected), moderate (25–50% affected) and severe (greater than 50% affected) and were converted into numeric scores of 0, 1, 2, 3, yielding a maximum possible score of 12 [44]. The CAA score was assessed in a similar fashion as none, mild, moderate and severe (0, 1, 2 and 3). The ApoE genotype was determined for each subject using a modification of the technique of Hixson and Vernier [50] as published previously [51].

### Immunohistochemistry

Formalin-fixed brain specimens were sectioned at 40 µm using a freezing stage microtome and stored at −20°C in a cryopreservative solution (30% glycerol+30% ethyleneglycol+20 mM phosphate buffer). All steps were performed at room temperature with washes in PBSTX (phosphate-buffered saline +0.3% Triton X-100) between each step unless otherwise indicated. Free-floating sections were placed in 1% H_2_O_2_ in PBSTX for 30 min and then blocked in 1% normal goat serum in PBSTX for 1 h. Sections were incubated in monoclonal mouse anti-human collagen IV primary antibody (Dako, Carpinteria, CA, cat. # 0785, dilution = 1∶1000) in PBSTX +1% bovine serum albumin+protease inhibitor cocktail (Roche) overnight, incubated in biotinylated goat anti-mouse IgG (Vector Laboratories, Burlingame, CA, dilution 1∶1000) in PBSTX with 1% normal goat serum for 2 h, followed by incubation in Strep-HRP (Invitrogen, Carlsbad, CA, SNN1004, dilution 1∶10,000) in PBSTX for 2 h. Sections were washed with PBSTX and Tris buffer (0.05 M Tris, pH 7.6) solutions. Staining was developed with nickel-enhanced 3,3′-diaminobenzidine for 30 min followed by washing with Tris buffer and the sections mounted on slides and dried. The slides were rehydrated in water, dehydrated by passage through a graded concentration alcohol solution series, cleared in xylene and mounted under a coverslip (Permount, Thermo Fisher Scientific, Waltham, MA). For vesicular acetylcholine transporter (VAChT) staining, the rabbit polyclonal anti-VAChT primary antibody (Phoenix Pharmaceuticals, Burlingame, CA, cat. # H-V005, dilution 1∶15,000) and a goat anti-rabbit-HRP secondary antibody (Jackson ImmunoResearch Laboratories, West Grove, PA) were used. For tyrosine hydroxylase (TH) staining, a rabbit polyclonal anti-TH primary antibody was employed (Millipore, Billerica, MA, cat. # AB152, dilution 1∶2000) and a biotinylated goat anti-rabbit was used as a secondary antibody (Vector Laboratories, cat. # BA-1000, dilution 1∶1000) followed by incubation with Strep-HRP as described above.

### Imaging Assessments

Collagen IV, VAChT and TH stained sections were imaged by systematic random sampling on a 4×4 mm grid. Imaging was performed with a Leica DMLB microscope using a Leica PLAN APO 10X objective lens (Buffalo Grove, IL) and captured with a Magnafire-SP camera (model s99805). Images from the entire section were obtained, but only those images that were clearly confined to either gray matter or white matter were analyzed. Images were analyzed using the National Institutes of Health image analysis software ImageJ (version 1.451: http://imagej.nih.gov/ij/). Using the Threshold tool in ImageJ, stained objects were selected and converted to image masks. To ensure that only capillaries were analyzed in the collagen IV images, larger diameter vessels were removed from the analysis manually. For accurate visual determination of the threshold for capillary images, the original images from collagen IV-stained sections were separated into red, green and blue channels in grayscale and only the red channel was analyzed as this yielded the best contrast between staining and the background. Similarly, the contrast was adjusted when analyzing VAChT images to visually select the point where threshold accuracy selected VAChT particles. The ImageJ Background Removal tool was used to reduce background color variations in TH images to enable accurate threshold selection. For capillary analysis, a total of 660 gray matter and 757 white matter field images were analyzed. For VAChT staining, 909 gray matter images were analyzed. For TH staining 710 gray matter and 757 white matter images were analyzed. All imaging and image analyses were done blinded.

### Analysis of Gray Matter Thickness

Slide-mounted sections were stained with filtered 1% Sudan black in 70% EtOH for 5 min then destained in 70% EtOH until the best contrast was obtained between gray and white matter. Slides were then coverslipped and scanned on a flatbed scanner. Images were analyzed in ImageJ. Approximately 100 measurements per case were made by using manual straight line measurements by selecting the shortest distance between the outer surface of the gray matter and the boundaries of the white matter over the entire ribbon of gray matter of the section. Since sections denote only a single plane, this may not represent a perpendicular projection relative to the gray matter surface. Therefore, it was assumed that the shortest distance corresponded to the most likely perpendicular plane. Thus, the lowest 30% of measurements were selected and averaged to estimate gray matter thickness for each case. Although the shortest dimensions gave a better estimate of gray matter thickness, inclusion of all measurements gave similar results.

### Statistical Analysis and BFI Calculation

Capillary density, brain mass and gray matter thickness, string vessel number and vascular innervation area were analyzed by one-way analysis of variance (ANOVA) and Tukey post hoc test, when applicable. Unpaired, 2-tailed t-tests were used to compare string vessel numbers in ApoE ε4 carriers and ApoE ε4 non-carriers and to compare ApoE ε3/3 individuals with and without plaques. Calculations were performed using GraphPad Prism 5 software (La Jolla, CA).

The BFI was calculated by using several neuropathological variables shown in **Table 1** and **Table S1** which included: brain weight, gray matter thickness, total NFT score, gray matter capillary number, gray matter capillary density, white matter capillary number, white matter capillary density, string vessel number, VAChT vesicle number, TH neurite density in gray matter, TH neurite density in white matter, total WMR score and total CAA score. All of these variables were converted into z-scores based on the mean and standard deviation of all non-AD individuals. The BFI represents the arithmetic mean of all the z-scores for each individual. For variables in which greater values indicate greater brain pathology (string vessels, total NFT score, total WMR, total CAA) the z-score was multiplied by -1 in order for the z-score to reflect the appropriate direction of effect. The z-scores of the OO-NPC and YO-NPC groups were almost identical and therefore combined into one NPC group to increase the statistical power of the control groups. ANOVA was used to discern group differences (AD vs. ND-HPC vs. NPC) on the BFI z-score. Bonferroni adjustment was used to correct for multiple group comparisons. The Kruskal-Wallis test was used to compare the BFI z-scores when individuals were grouped according to Braak stage (Group 1: ≤IV, Group 2: ≥V). Two-sample t-tests were used to determine if the BFI z-score differed significantly between males and females and also between ApoE ε4 carriers and non-carriers.

## Results

**Figure 1:**
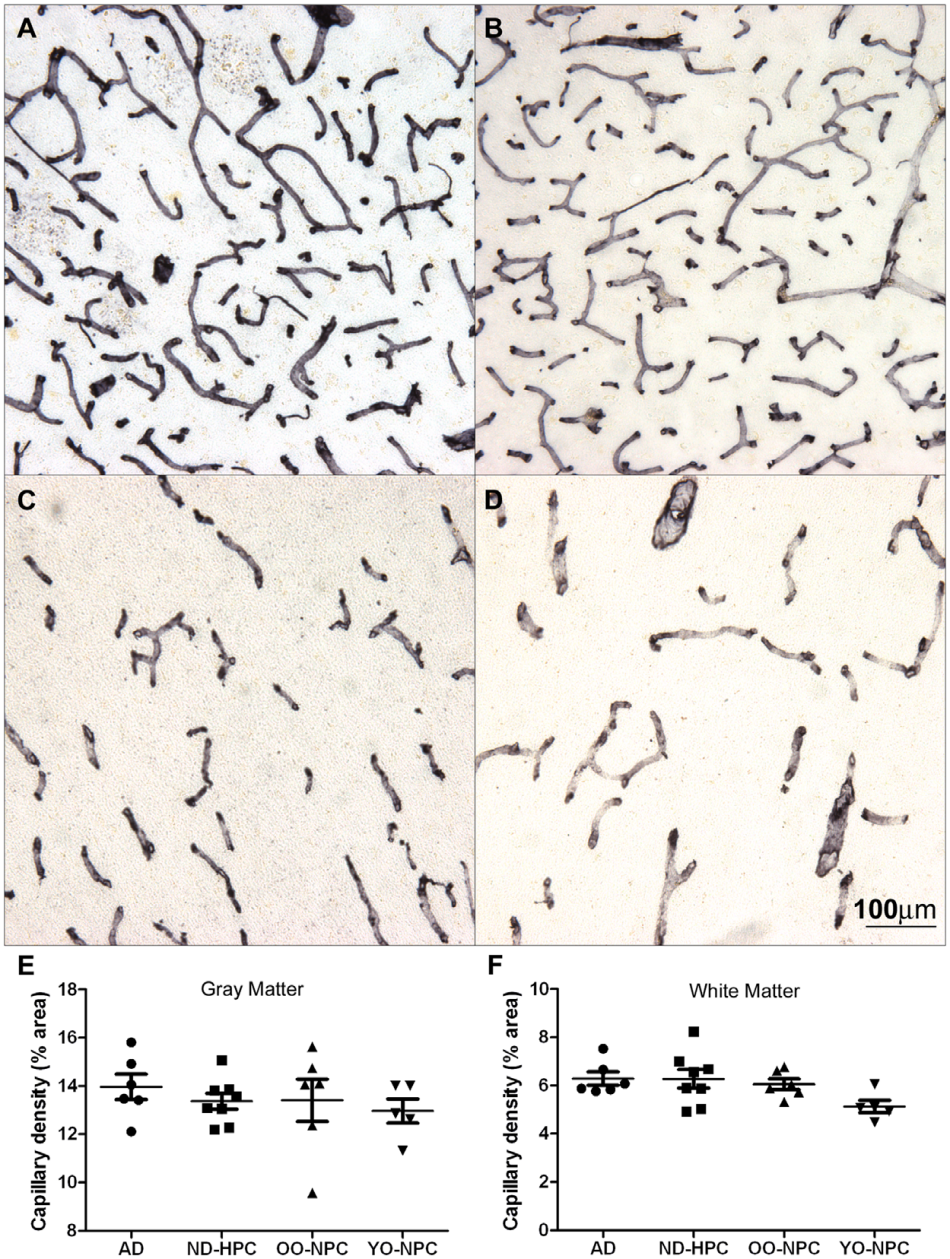
Capillary density in gray matter and white matter. Sections from all cases were stained with collagen IV. A) Gray matter image from an AD case. B) Gray matter image from a YO-NPC case. C) White matter image from an ND-HPC case. D) White matter image from an OO-NPC case. E-F) Capillary density was determined by image analysis using ImageJ software as described in the Materials and Methods section. Each data point represents the average % area covered by capillaries in numerous images from a single case. Bars represent the average of all cases and error bars represent the SEM for all cases in a group. E) Gray matter capillary density. F) White matter capillary density. Groups were not significantly different as determined by One-way ANOVA. The scale bar is applicable to all images in Figure 1.

**Figure 2:**
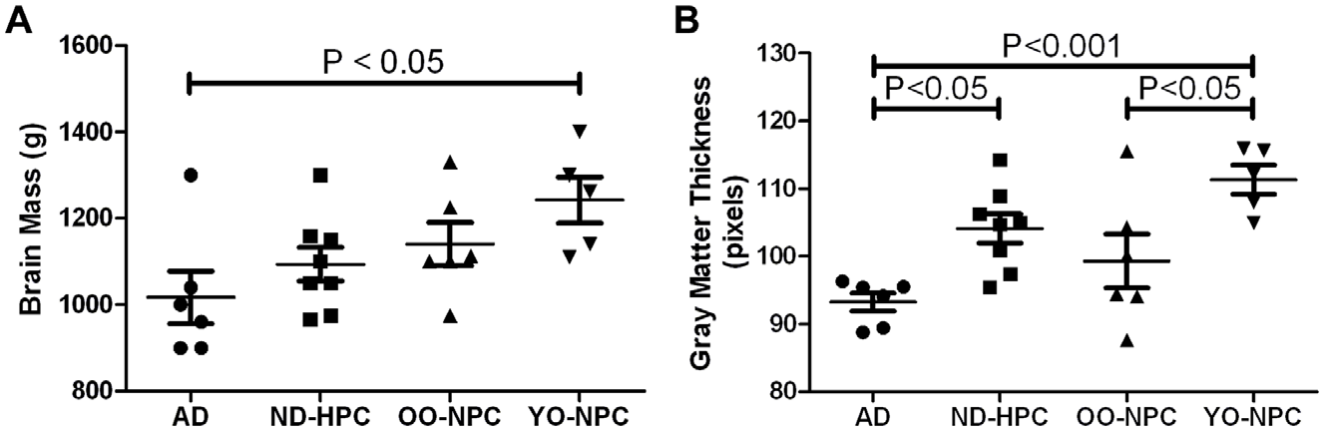
Brain mass and gray matter thickness. A) Total brain mass was determined at the time of autopsy for each case. B) Gray matter thickness for each case was determined by numerous straight-line measurements of the gray matter ribbon on sections from each case. Represented in each data point is the average of the lowest 30% of measurements for each case. In graphs A and B, bars represent the average for all cases in the group and error bars represent the SEM. Statistical analysis: One-way ANOVA was performed for brain mass (*p* = 0.047) and for gray matter thickness (*p* = 0.0012). A Tukey post test was performed for pair-wise comparison of all groups and significance is indicated on the graphs.

In order to investigate the integrity of the microvasculature in AD and ND control groups, brain sections were stained with collagen IV **(** **Figure 1A–D** **)** and the total capillary density for each case was determined **(** **Figure 1E–F** **)**. The average gray matter capillary density was highest in the AD group and lowest in the YO-NPC group, although these differences did not attain a level of statistical significance **(** **Figure 1E** **)**. Results were similar in the white matter in which all nonagenarian groups were nearly identical, with the YO-NPC group having the lowest capillary density **(** **Figure 1F** **)**. While capillary density was not significantly altered, total brain mass, determined at autopsy, was significantly less in AD compared to ND control groups **(** **Figure 2A** **)**. Quantifying cortical thickness on Sudan black stained sections revealed the gray matter in AD cases was 16% thinner than YO-NPC cases **(** **Figure 2B** **)**.

**Figure 3:**
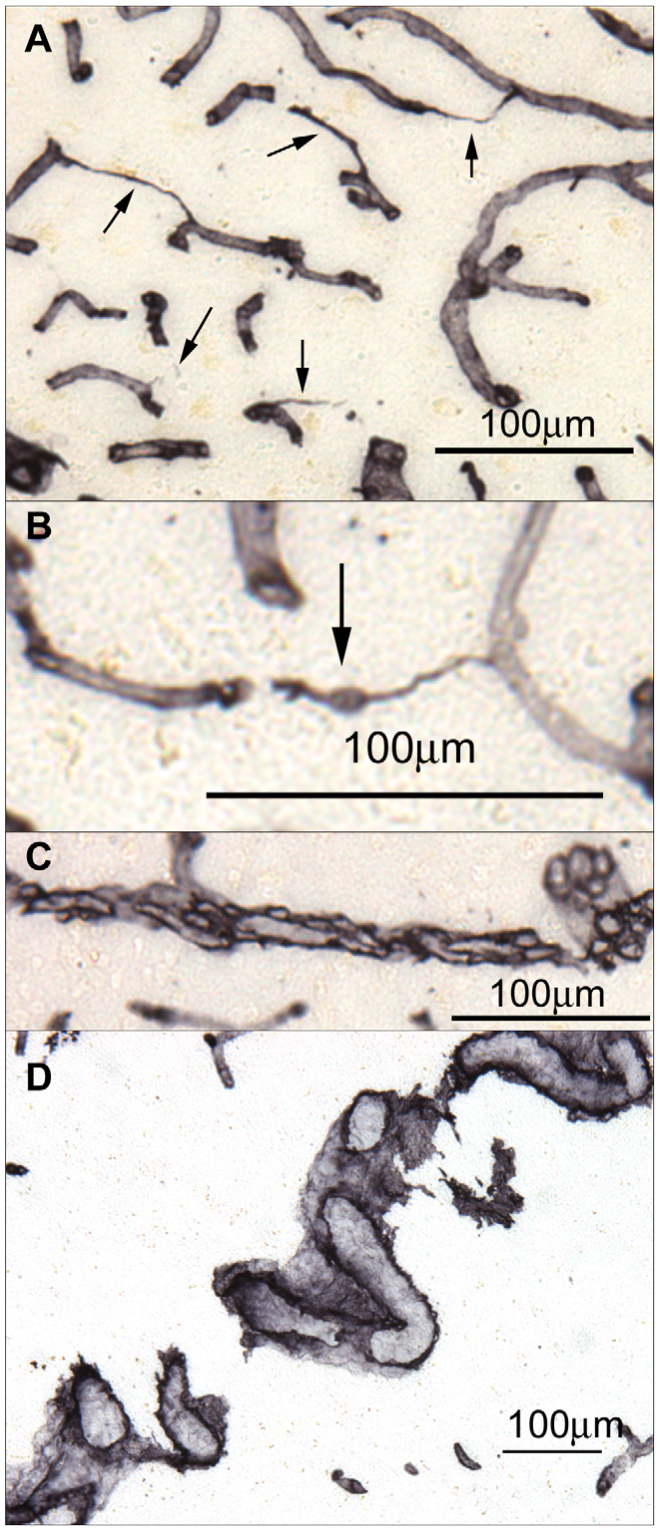
Blood vessel abnormalities in the oldest-old. A) An area of high string vessel density in an AD case. String vessels are indicated by arrows. B) A string vessel exhibiting a microembolus (arrow) in an AD case. C) Abnormally bundled capillaries in an AD case. D) A large tortuous vessel in the white matter of an AD case. All sections were stained for collagen IV.

**Figure 4:**
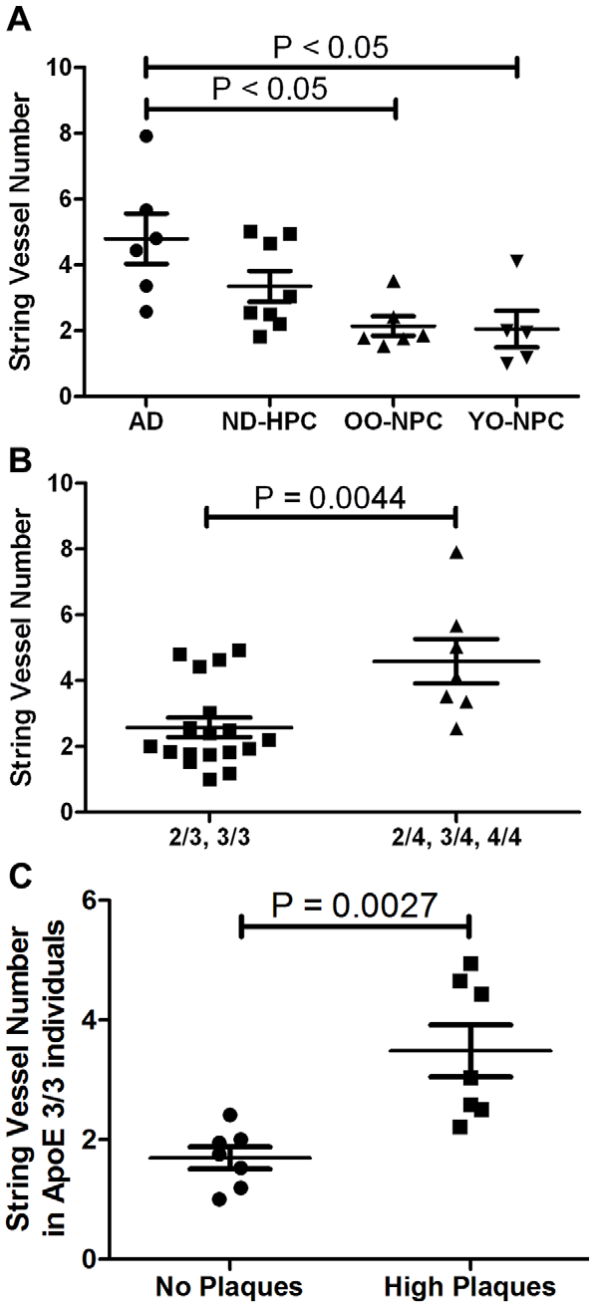
Distribution of string vessels in the four studied groups. A) The number of string vessels was determined by counting string vessels in collagen IV-stained sections. Each data point represents the average number of string vessels per field for numerous images for each case. Bars represent the average of all cases in a group and error bars represent the SEM. Statistical analysis: One-way ANOVA revealed statistically increased string vessel numbers in AD compared to ND control groups; *p* = 0.0076, with a Tukey post test indicating statistical increase in AD compared to OO-NPC and YO-NPC groups. B) String vessel number stratified according to ApoE ε4 carrier status. Statistical analysis: t-test reveals a significant increase in string vessels in ApoE ε4 carriers (*p* = 0.0044). C) String vessel number of ApoE ε3/3 individuals with or without amyloid plaques. Statistical analysis: t-test reveals a significant increase in string vessel number in cases with amyloid plaques (*p* = 0.0027).

Blood flow shear stress is a critical factor for maintaining vascular endothelial cell survival. Decreased blood flow and/or occlusion by microemboli contribute to loss of shear stress, endothelial cell death, collapse of capillaries and the formation of string vessels **(** **Figure 3A** **)**. Infrequent evidence of microembolism **(** **Figure 3B** **)** and sporadic bundled capillaries **(** **Figure 3C** **)** as well as occasional tortuous vessels of larger caliber **(** **Figure 3D** **)** were also observed. On average, the AD gray matter had more than double the number of string vessels found in OO-NPC and YO-NPC groups while the ND-HPC group was intermediate **(** **Figure 4A** **)**. Intriguingly, when stratified according to ApoE genotype alone, ApoE ε4 carriers had significantly higher string vessel counts relative to non-ε4 carriers regardless of cognitive status **(** **Figure 4B** **)**. When stratifying only by the presence or absence of amyloid plaques and including only ApoE ε3/3 individuals, those with plaques had significantly higher string vessel counts than those without plaques **(** **Figure 4C** **)**. It is important to note that string vessels were included in the capillary density analysis while vessels larger than capillaries were excluded.

**Figure 5:**
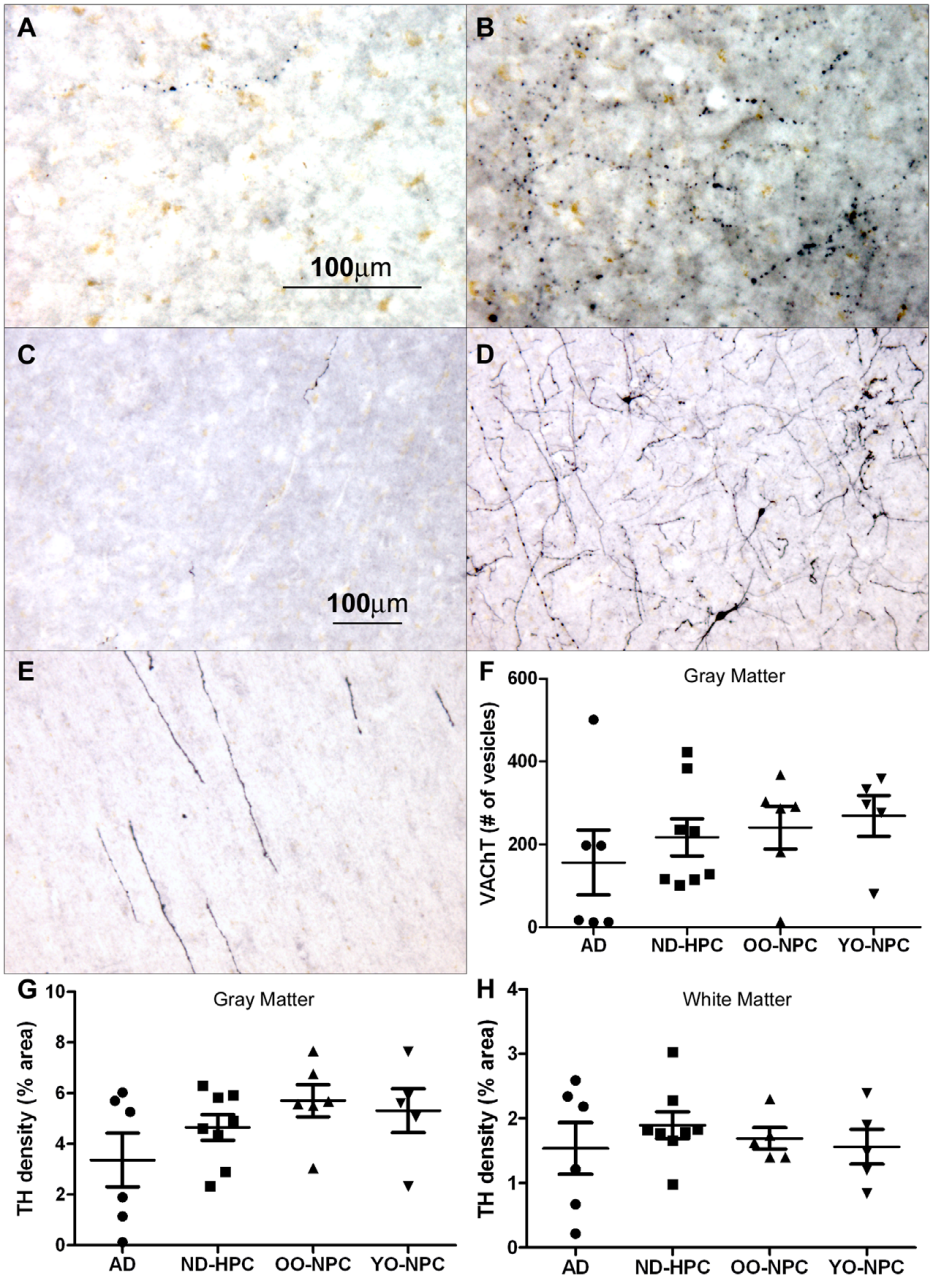
Vascular innervation of the oldest-old. A-B) The amount of innervation of the cortex by cholinergic neurons was determined by staining with VAChT. A) An example of low VAChT staining in an AD case. B) An example of high VAChT staining in a YO-NPC case. C-D) The amount of innervation of the cortex by noradrenergic neurons was determined by staining with TH. C) An example of low TH staining in AD case. D) An example of high TH staining in a YO-NPC case gray matter. E) White matter TH staining in an OO-NPC. F) Cholinergic innervation of cortical gray matter was quantified by counting the number of VAChT-positive vesicles per field using ImageJ software as described in the Materials and Methods section. Each point represents the average number of vesicles observed in each case. G) Noradrenergic innervation of cortical gray matter, as determined by TH staining, was quantified by determining the % area stained in each image. Each point represents the average % area observed in each case. H) The TH immunoreactivity in cortical white matter was quantified as in the gray matter. For all graphs, bars represent the average of all cases for each group with their SEM. Groups were not significantly different by One-way ANOVA. Magnification: A and B = 200X; C, D and E = 100X.

Brain sections were stained for VAChT **(** **Figure 5A–B** **)** and TH **(** **Figure 5C–E** **)** in order to investigate whether or not loss of cholinergic and noradrenergic innervations contribute to AD pathology, respectively. Examples of the range of VAChT and TH densities are depicted in **Figures 5A–E**. VAChT-positive vesicles were quantified in the gray matter and a trend was found toward reduced VAChT particle number in AD compared to ND control groups **(** **Figure 5F** **)**. However, the difference did not reach the level of statistical significance due to high individual variability. Three AD cases had virtually no VAChT and on the average, the YO-NPC group had about twice as many VAChT-positive vesicles than the AD group **(** **Figure 5F** **)**. Decreased TH staining was found in grey matter and white matter in AD cases compared to ND control cases although these differences did not reach statistical significance **(** **Figure 5G and 5H** **,** respectively**)**. In the AD group, there were three cases with very little TH immunoreactivity in the grey matter and three that had similar levels of staining as the ND controls **(** **Figure 5G** **)**. Assessment of the TH-positive neurites in the white matter also yielded similar results **(** **Figure 5H** **)**. Importantly, the levels of TH and VAChT staining were positively correlated (correlation coefficient 0.732).

**Figure 6:**
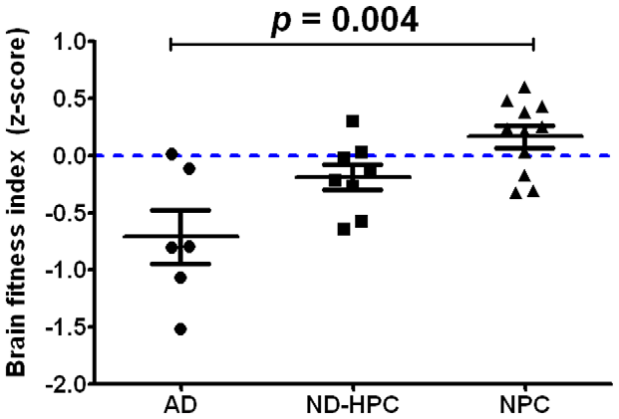
Brain fitness index. The overall brain fitness of each individual was determined by statistical analysis of 13 parameters described in Materials and Methods section and shown in **Table 1** and **Table S1**. The BFI represents the mathematical average of all the z-scores for each individual. Bars represent the average of all cases for each group with error bars representing the SEM. Statistical analysis: One-way ANOVA demonstrated statistical difference (F = 7.21 df = (2, 22), *p* = 0.004). Bonferroni adjustment found that the AD and ND-HPC groups were not significantly different (*p* = 0.06) and that the AD and NPC groups were significantly different (*p* = 0.003). The ND-HPC and NPC groups were not significantly different (*p* = 0.73). Both AD and ND-HPC had a negative BFI relative to the NPC group, being more negative in the AD group.

A statistical analysis was performed by combining the neuropathological findings (see **Table 1** and **Table S1**) from each case to calculate the BFI. The individual BFI z-scores for all study participants are displayed in **Figure 6**. The NPC group had higher BFI z-scores and lower variability among their scores than the other two groups. However, there was some degree of overlap on the BFI z-scores between groups. Overall, there was a statistically significant effect for the BFI (F = 7.21, df = (2,22), *p* = 0.004). Bonferroni adjustment found that the AD and NPC groups were significantly different from each other (*p* = 0.003), but the difference between AD and ND-HPC groups fell just short of significance (*p* = 0.06) for the BFI z-score. The ND-HPC and NPC groups were not significantly different (*p* = 0.73) from each other. An additional analysis comparing the BFI z-scores by Braak stage was also carried out. Individuals were classified into two groups based on those who were at or below Braak Stage IV (n = 20) and those who were above Braak stage V (n = 5). Due to the imbalance in group sizes, the non-parametric Kruskal-Wallis test was carried out and found that the lower Braak stage group had significantly higher BFI z-scores than the higher Braak stage group (Kruskall-Wallis = 7.76, p = 0.005). A separate ANOVA was carried out to compare the string vessel count between groups. It was found that only the AD and NPC groups were significantly different from each other after adjusting for multiple comparisons (F = 8.19, (df = 2,22), *p* = 0.002). The ND-HPC group was not significantly different from either the AD or NPC groups. Among the entire study sample, there was no significant difference on the BFI z-score between males and females (t = −0.67, df = 23, *p* = 0.53). In addition, there was no significant difference between ApoE ε4 carriers and non-carriers (t = 1.07, df = 23, *p* = 0.30).

**Figure 7:**
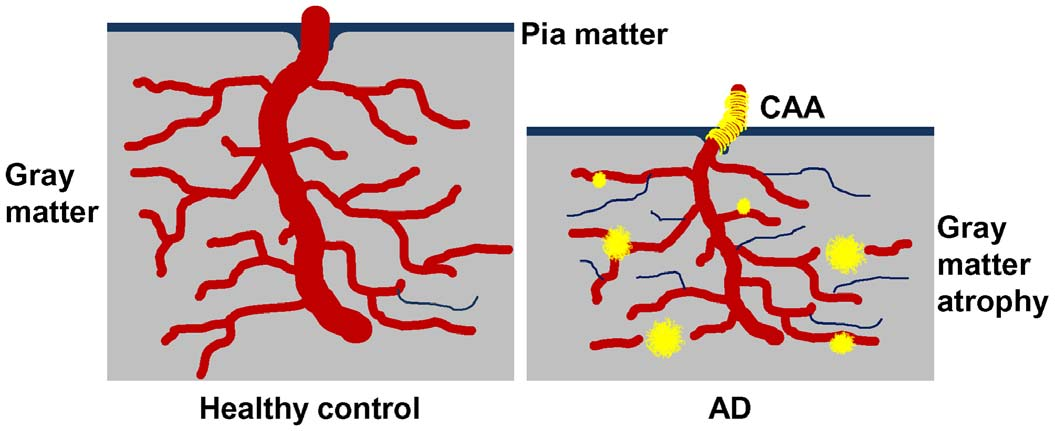
A representation of microvascular compromise in AD. On the left is a representation of the cerebral cortex of an individual with an optimal vasculature and efficient cerebral blood flow with no amyloid plaques, sparse string vessels and no gray matter atrophy. On the right is a depiction of pathological condition in which the capillary density is relatively maintained, but with decreased cerebral blood flow resulting from numerous string vessels. Whether string vessels are the cause or effect of grey matter hypoperfusion and eventual brain atrophy is unknown. However, evidence suggests that parenchymal plaques may initially be associated with capillaries and arterioles in an attempt to seal microvascular leakage [74]. At more advanced stages, the pressure exerted by the growing perivascular amyloid deposits constricts the microvessel, leaving dysfunctional capillary stumps. In addition, amyloid deposits associated with larger cortical and leptomeningeal vessels ultimately destroy vascular smooth muscle and endothelial cells. Brain perfusion is further damaged by a compromised interstitial fluid drainage due to destruction of the perivascular spaces [25].

## Discussion

Our observations revealed that, in the oldest-old, the overall capillary density in AD gray matter and white matter was not significantly decreased compared to the ND control groups. It is possible that since the capillary density relative to brain volume is approximately the same among the groups, the rate of capillary loss could have advanced at similar rates. In fact, we are faced with a dilemma in which we do not know if capillary loss was responsible for brain volume loss, or if brain volume loss led to capillary demise or if both brain atrophy and capillary paucity proceeded at the same time. Although controversial, the majority of the morphometric studies report a decrease in vascular density in AD as reviewed by Brown et al. [26]. Brown’s observations also show that in the deep white matter in younger AD cases, the density of microvessels is lower than controls. However, vascular density decreases with age in normal controls as well, and converges with the AD cases by the 9th decade of life [52].

Variations in age, disease stage, brain region and methodology for quantifying vascular density could also explain the diversity of results among the different studies. However, similarity in microvascular density does not necessarily translate into functional equality. For example, there is significant physical evidence for decreased cerebral perfusion in AD using MRI imaging and ultrasound technologies [19], [34], [53], [54]. These hemodynamic observations strongly support the contention that in AD there is diffuse microvascular disease.

While capillary density was not significantly decreased, there was an overall brain atrophy and thinning of cortical grey matter in AD compared to ND control groups. Grey matter thickness was slightly reduced in OO-NPC relative to ND-HPC although not significantly, but was clearly decreased in AD cases. This may indicate that the grey matter cannot be reduced too severely without resulting in cognitive decline and moving into the AD category.

Decreased cerebral blood flow along with the findings of similar capillary densities in the current study, may be explained by the presence of string vessels. String vessels may form as a result of endothelial cell death followed by collapse of the capillary walls, leaving only remnants of the extracellular matrix in the form of a thin string. It is thought that shear stress caused by blood flow is required for vascular endothelial cell survival and continued vessel patency. Loss of shear stress can be caused by at least two mechanisms; loss of blood flow or vascular blockage by microemboli that are not extravasated [26], [27], [55], [56]. While some string vessels were likely the result of blockage by microemboli, these events were uncommon, suggesting a general loss of blood flow as the more typical cause of string vessel formation.

The number of string vessels in the YO-NPC group was virtually identical to the OO-NPC group, thereby distinguishing these groups from the AD group. The average number of string vessels in the ND-HPC was lower than in the AD group, but higher than the NPC groups demonstrating an intermediate, but clear, disruption of the microvasculature. The fact that ND individuals survived into the 9^th^ decade of life suggests a better “cognitive reserve” or microvasculature quality, which may result in enhanced brain perfusion. The data support the need for understanding the processes that control microvascular density, capillary loss, angiogenesis [57] and string vessel genesis [27], all in the context of atrophic grey and white matter [58]. All of these observations support the hypothesis of decreased perfusion in AD and could explain the increased number of string vessels observed in this study. While it has been reported that string vessel numbers are elevated in AD [26], [59], no study has focused on AD cases of such advanced age and on the unique ND control groups used in this study.

The presence of ApoE ε4 correlates with string vessel number. Likewise, the numbers of string vessels also appeared to be associated independently with the number of amyloid plaques. Unfortunately, ApoE status and amyloid load could not be tested together as predictors of string vessel numbers, since we have a small sample size and total amyloid plaque score was used as sample selection criteria. It has been hypothesized that amyloid plaques form as a result of capillary leakage and that plaques may eventually cut off blood flow leaving blind capillary stumps [60]–[62]. In addition, Aβ has been shown to have anti-angiogenic properties [63], [64]. In light of recent findings of increased angiogenesis in AD, Aβ may be released to control aberrant angiogenesis [65]. In exudative macular degeneration, vascular endothelial growth factor (VEGF) expression, induced by hypoxia, drives anomalous angiogenesis and these new vessels leak resulting in the formation of Aβ-positive plaques. This explanatory chain of events was recently reinforced by the effectiveness of anti-Aβ antibodies in the treating macular degeneration [66], [67].

The physical nature and rapid development of individual amyloid plaques [68] suggests a swift response to a focal insult such as a leaky capillary, or CAA formation around larger vessels whose permeability may be compromised. While it is tempting to think that string vessels follow occlusion by compact amyloid plaque cores, this may not be the case. It is feasible that two independent mechanisms could be in play. For instance, reduced perfusion and the resultant hypoxia could induce angiogenesis and formation of amyloid plaques at the site of new leaky capillaries, while simultaneously, loss of blood flow and shear stress would result in the formation of string vessels at different loci. Additional studies regarding the relationship between string vessels and amyloid plaques are warranted.

One mechanism of regulating regional cerebral blood flow is by neuronal modulation of vasodilatation and vasoconstriction. Cholinergic afferents from the NBM project to the cerebral cortex to regulate vasodilation mediated by a nitric oxide synthase response. Vasoconstriction is in part regulated by the noradrenergic locus ceruleus (LC) [41]. In AD, one of the nuclei first affected in the course of this dementia is the NBM [69] and examination of both NBM and LC in AD revealed a marked depletion of neurons [70], [71]. While not statistically significant, we found that on average, AD cases had less innervation as determined by VAChT and TH immunohistochemistry, suggesting that this depletion may contribute to the vascular lesions demonstrated in this paper. Ablation of the nucleus basalis magnocellularis in the rabbit (corresponding to NBM in humans) results in the deposition of perivascular Aβ in cortical microvessels demonstrating a critical link between Aβ pathology and the neurovascular unit [38]. Interestingly, loss of VAChT innervation also correlated well with loss of TH staining, but not other dysfunctional parameters, suggesting that these two markers are mechanistically independent from other pathologies.

To better estimate the overall brain health of the individuals in our study, we created a BFI-z score. This index revealed that the NPC cohorts in our study had the best BFI-z scores and served as a frame of ‘healthy’ reference, while AD cases had many deleterious contributing factors. On the average, the ND-HPC group had an intermediate BFI suggesting that they may have had prodromal AD or other compensatory mechanisms that protected them from dementia. While many of the parameters in our BFI were determined postmortem, similar multifaceted approaches are beginning to be adopted using many biomarkers from imaging, CSF, plasma and other physical measurements in the living to better estimate the risk of dementia and rate of decline [72]. The ApoE ε4 risk was not a significant contributing factor to the BFI in our study and thus was not included in the BFI calculation. This is likely due to several factors such as small sample size or to the decreasing ApoE ε4 risk factor effect with advancing age [73]. Taken together the data suggest that while a high amyloid plaque burden may be present in an individual, as in our ND-HPC group, cognitive status and brain fitness depend on many other factors.

In summary, our data reveal microvascular morphological alterations and loss of vascular innervation, supporting the hypothesis that hemodynamic changes may contribute to VCI and dementia. Further, in spite of high amyloid plaque numbers, some nonagenarian individuals survive without dementia eliminating these lesions as the sole factor in AD pathogenesis. In the end, it remains unclear whether a decrease in brain perfusion is responsible for neurodegeneration or if the primary loss of neurons, neurites and synapses is accountable for poor brain perfusion or both. Nevertheless, we suggest that multiple, concurrent cardiovascular and cerebrovascular pathological factors decrease blood flow. Consequential brain hypoperfusion results in neurovascular unit functional impairment and microvascular demise **(** **Figure 7** **)**. Neurons in proximity to string vessels and amyloid plaques eventually perish due to localized hypoxia/oligemia, thus contributing to the cognitive deficits seen in AD. Lifestyle changes and timely pharmacological interventions that promote vascular health and enhance perfusion may therefore be beneficial in preventing or slowing the progression of AD.

## Supporting Information

Table S1 **Abbreviations and explanations.** AD = Alzheimer’s disease; ND-HPC = non-demented high pathology control; OO-NPC = oldest-old no plaque control; YO = young-old no plaque control; ID = Case identification number, y = years. String vessels indicates the average number of string vessels from numerous images, TH = tyrosine hydroxylase and TH density indicates the average % area of numerous images of sections stained with an anti-TH antibody. VAChT = vesicular acetylcholine transporter and VAChT vesicles indicates the average number of vesicles per image from numerous images of VAChT stained sections. Gray matter thickness is the distance in pixels from the tissue edge to the nearest area of white matter. Capillary number represents the average number of capillary objects from numerous images of collagen IV stained sections while capillary density represents the average % area covered by capillaries from the same images.
(DOCX)
