# Supplementary material for: Morphological and Pathological Evolution of the Brain Microcirculation in Aging and Alzheimer’s Disease
Source: PLoS One. 2012 May 16;7(5):e36893. doi: 10.1371/journal.pone.0036893 (PMC3353981; doi:10.1371/journal.pone.0036893)
Supplement: Table S1 — Abbreviations and explanations. AD = Alzheimer’s disease; ND-HPC = non-demented high pathology control; OO-NPC = oldest-old no plaque control; YO = young-old no plaque control; ID = Case identification number, y = years. String vessels indicates the average number of string vessels from numerous images, TH = tyrosine hydroxylase and TH density indicates the average % area of numerous images of sections stained with an anti-TH antibody. VAChT = vesicular acetylcholine transporter and VAChT vesicles indicates the average number of vesicles per image from numerous images of VAChT stained sections. Gray matter thickness is the distance in pixels from the tissue edge to the nearest area of white matter. Capillary number represents the average number of capillary objects from numerous images of collagen IV stained sections while capillary density represents the average % area covered by capillaries from the same images. (DOCX) [file pone.0036893.s001.docx]

**Table 1S: Tabulation of microvascular staining and immunohistochemistry results.**

| ID | Expired age (y) | String vessels (gray) | TH density (gray) | TH density (white) | VAChT vesicles (gray) | Gray matter thickness | Capillary number  (gray) | Capillary density  (gray) | Capillary number  (white) | Capillary density  (white) | Brain fitness index |
| --- | --- | --- | --- | --- | --- | --- | --- | --- | --- | --- | --- |
| AD |  |  |  |  |  |  |  |  |  |  |  |
| 10 | 95 | 5.67 | 6.03% | 2.34% | 501 | 95.4 | 88.6 | 13.5% | 33.9 | 7.53% | -0.11 |
| 11 | 90 | 4.44 | 1.15% | 0.67% | 17 | 88.8 | 87.2 | 13.4% | 34.9 | 6.08% | -0.79 |
| 12 | 96 | 2.58 | 5.26% | 2.59% | 197 | 96.3 | 114.0 | 12.1% | 38.6 | 5.88% | 0.01 |
| 13 | 96 | 4.80 | 5.70% | 2.18% | 197 | 94.2 | 101.0 | 15.8% | 30.3 | 5.76% | -0.80 |
| 14 | 96 | 7.91 | 1.90% | 1.21% | 12 | 89.4 | 89.0 | 14.9% | 27.5 | 5.83% | -1.51 |
| 15 | 92 | 3.36 | 0.12% | 0.22% | 13 | 95.5 | 90.6 | 14.1% | 29.5 | 6.66% | -1.06 |
| Mean | 94.2 | 4.79 | 3.36% | 1.53% | 156 | 93.3 | 95.1 | 14.0% | 32.5 | 6.29% | -0.71 |
| ND-HPC |  |  |  |  |  |  |  |  |  |  |  |
| 1 | 91 | 5.03 | 4.91% | 1.66% | 117 | 114.3 | 94.7 | 15.1% | 37.5 | 8.24% | 0.30 |
| 2 | 100 | 2.21 | 5.83% | 1.82% | 423 | 105.0 | 88.6 | 12.2% | 27.7 | 5.03% | -0.27 |
| 3 | 90 | 2.50 | 6.30% | 1.79% | 384 | 104.7 | 90.2 | 13.1% | 28.0 | 5.90% | 0.03 |
| 4 | 94 | 4.66 | 2.34% | 1.77% | 236 | 95.5 | 100.7 | 13.9% | 42.8 | 6.56% | -0.57 |
| 5 | 90 | 3.04 | 5.93% | 2.28% | 102 | 106.3 | 80.9 | 13.8% | 31.9 | 7.01% | -0.15 |
| 6 | 92 | 2.55 | 2.91% | 0.98% | 115 | 97.4 | 99.1 | 12.3% | 30.8 | 4.93% | -0.64 |
| 7 | 91 | 1.83 | 4.37% | 1.83% | 232 | 108.9 | 90.5 | 13.1% | 33.2 | 5.88% | -0.02 |
| 8 | 94 | 4.95 | 4.61% | 3.03% | 129 | 100.9 | 87.4 | 13.6% | 35.1 | 6.69% | -0.22 |
| Mean | 92.8 | 3.34 | 4.65% | 1.89% | 217 | 104.1 | 91.5 | 13.4% | 33.4 | 6.28% | -0.19 |
| OO-NPC |  |  |  |  |  |  |  |  |  |  |  |
| 50 | 91 | 1.86 | 3.04% | 1.40% | 14 | 104.3 | 83.3 | 12.4% | 28.0 | 5.88% | -0.33 |
| 51 | 91 | 3.51 | 7.66% | 2.30% | 368 | 115.5 | 85.4 | 14.1% | 36.3 | 5.72% | 0.60 |
| 52 | 91 | 1.78 | 5.50% | 1.62% | 303 | 94.4 | 91.5 | 14.1% | 32.9 | 6.61% | 0.23 |
| 53 | 91 | 1.76 | 6.76% | -- | 181 | 100.1 | 90.7 | 14.7% | 41.0 | 6.77% | 0.43 |
| 54 | 99 | 2.41 | 5.68% | 1.73% | 291 | 94.1 | 94.9 | 15.6% | 34.3 | 6.04% | 0.21 |
| 55 | 92 | 1.53 | 5.57% | 1.40% | 287 | 87.6 | 82.7 | 9.6% | 33.0 | 5.32% | -0.31 |
| Mean | 92.5 | 2.14 | 5.70% | 1.69% | 241 | 99.3 | 88.1 | 13.4% | 34.2 | 6.06% | 0.14 |
| YO-NPC |  |  |  |  |  |  |  |  |  |  |  |
| 60 | 75 | 4.11 | 5.06% | 1.48% | 358 | 115.9 | 82.4 | 12.6% | 31.5 | 5.07% | 0.03 |
| 61 | 71 | 1.19 | 5.61% | 1.90% | 296 | 115.6 | 98.2 | 12.8% | 26.2 | 4.48% | 0.48 |
| 62 | 68 | 1.00 | 5.90% | 2.39% | 276 | 105.0 | 76.8 | 14.0% | 30.7 | 6.05% | 0.25 |
| 63 | 65 | 2.00 | 7.64% | 1.20% | 333 | 112.1 | 76.7 | 14.0% | 29.6 | 5.10% | 0.38 |
| 64 | 75 | 1.95 | 2.32% | 0.84% | 80 | 108.0 | 99.6 | 11.3% | 30.7 | 4.94% | -0.17 |
| Mean | 70.8 | 2.05 | 5.30% | 1.56% | 269 | 111.3 | 86.7 | 13.0% | 29.7 | 5.13% | 0.19 |
